# A model-based estimate of winter distribution and abundance of white-tailed deer in the Adirondack Park

**DOI:** 10.1371/journal.pone.0273707

**Published:** 2022-08-30

**Authors:** Joseph W. Hinton, Jeremy E. Hurst, David W. Kramer, James H. Stickles, Jacqueline L. Frair

**Affiliations:** 1 Department of Environmental and Forest Biology, State University of New York College of Environmental Science and Forestry, Syracuse, New York, United States of America; 2 Division of Fish and Wildlife, New York State Department of Environmental Conservation, Albany, New York, United States of America; 3 Roosevelt Wild Life Station, State University of New York College of Environmental Science and Forestry, Syracuse, New York, United States of America; US Geological Survey, UNITED STATES

## Abstract

In the Adirondack Park region of northern New York, USA, white-tailed deer (*Odocoileus virginianus*) and moose (*Alces alces*) co-occur along a temperate-boreal forest ecotone. In this region, moose exist as a small and vulnerable low-density population and over-browsing by white-tailed deer is known to reduce regeneration, sustainability, and health of forests. Here, we assess the distribution and abundance of white-tailed deer at a broad spatial scale relevant for deer and moose management in northern New York. We used density surface modeling (DSM) under a conventional distance sampling framework, tied to a winter aerial survey, to create a spatially explicit estimate of white-tailed deer abundance and density across a vast, northern forest region. We estimated 16,352 white-tailed deer (95% CI 11,762–22,734) throughout the Adirondack Park with local density ranging between 0.00–5.73 deer/km^2^. Most of the Adirondack Park (91.2%) supported white-tailed deer densities of ≤2 individuals/km^2^. White-tailed deer density increased with increasing proximity to anthropogenic land cover such as timber cuts, roads, and agriculture and decreased in areas with increasing elevation and days with snow cover. We conclude that climate change will be more favorable for white-tailed deer than for moose because milder winters and increased growing seasons will likely have a pronounced influence on deer abundance and distribution across the Adirondack Park. Therefore, identifying specific environmental conditions facilitating the expansion of white-tailed deer into areas with low-density moose populations can assist managers in anticipating potential changes in ungulate distribution and abundance and to develop appropriate management actions to mitigate negative consequences such as disease spread and increased competition for limiting resources.

## Introduction

Given that climate change and anthropogenic alterations to landscapes threaten the persistence of many species, there is a growing need for landscape-scale research and monitoring to investigate relationships between species abundance and landscape characteristics. Understanding key wildlife-habitat relationships is necessary for implementing effective management to minimize the negative impacts that climate change will have on cold-adapted species, particularly on populations experiencing declines along the southern edges of their species ranges. For example, warming winters caused by climate change can affect specific physiological (i.e., thermoregulation) and biotic (i.e., competition and epizootics) processes known to influence the abundance and distribution of ungulates [1–3]. When ungulates respond to changing conditions on the landscape, they shift their population distribution in response to the distribution of favorable conditions [4, 5].

Shifts in the distribution of ungulates, such as white-tailed deer (*Odocoileus virginianus*), in the northern forests of North America are attributed to decreased severity of winter conditions, reduced snow cover, and extended growing seasons [1, 4, 6, 7]. However, white-tailed deer exhibit chronic winter mortality at northern latitudes, so they respond to severe winter weather and deep snow by congregating in wintering areas known as deer yards [8–12]. These areas are typically characterized by forest types offering shelter and browse (e.g., coniferous forests), low elevation, and contain a network of corridors used by deer for movement and foraging. Warming winters associated with climate change have reduced overwinter mortality of white-tailed deer in areas along their northern range and facilitated increased deer presence in northern forests well above their historical levels [4]. Increasing densities of white-tailed deer in northern forests are known to support greater densities of large predators [13–15] and facilitate the spread of parasitic pathogens [16–19] that have negative consequences for other ungulates, such as elk (*Cervus canadensis*), moose (*Alces alces*), and woodland caribou (*Rangifer tarandus caribou*). Therefore, quantifying the influence of land cover on the distribution and abundance of white-tailed deer is essential for anticipating future changes in ungulate numbers and for informing management plans to mitigate the negative consequences of increasing deer densities.

In the Adirondack Park region (hereafter “the Park”) of northern New York, white-tailed deer and moose co-occur along an ecotonal temperate-boreal forest region where deer are expanding their range [20] and moose exist as a stable, low-density population [21]. Mild winters associated with climate change have allowed white-tailed deer to winter in land cover that formerly could only sustain them in the summer [3, 4]. White-tailed deer in the Park respond positively to anthropogenic landscape features by concentrating their winter yards along corridors of residential development and roads for shelter, forage, and movement corridors during winter [12]. Moose in the Park are non-migratory and most of the low-density population inhabits the northern region where large-scale forestry operations provide concentrated patches of regenerative forest [21]. Both species rely on timber harvest forest regeneration for forage and ongoing timber management in the northern areas of the Park has the potential to further concentrate moose and white-tailed deer in that region. Additionally, warmer winters will increase transmission risks for parasitic pathogens and threaten long-term persistence of moose in the region. Therefore, given the complexity of how white-tailed deer exploit land cover and the impact of increasing deer densities on moose recovery in the Park, assessing the distribution and abundance of deer at a broad spatial scale is relevant for deer management that requires integrating information across different spatial scales.

Our primary objective was to assess the winter population size and distribution of white-tailed deer in the Park using the 2-stage density surface modeling (DSM) approach [22]. This entailed developing spatially explicit models of white-tailed deer abundance in the Park by coupling conventional distance sampling (CDS) with multivariate regression modeling to produce a density surface map (deer/km^2^) predicted from environmental covariates [22, 23]. The DSM approach uses the spatial variability of white-tailed deer abundance to model abundance at finer resolutions than CDS alone, provide additional insight into species-environment relationships within survey areas, and predict abundance in neighboring areas that were not surveyed. Our secondary objective was to compare winter distribution and density of white-tailed deer in the Park to those previously estimated for moose [21].

## Material and methods

### Study area

**Fig 1:**
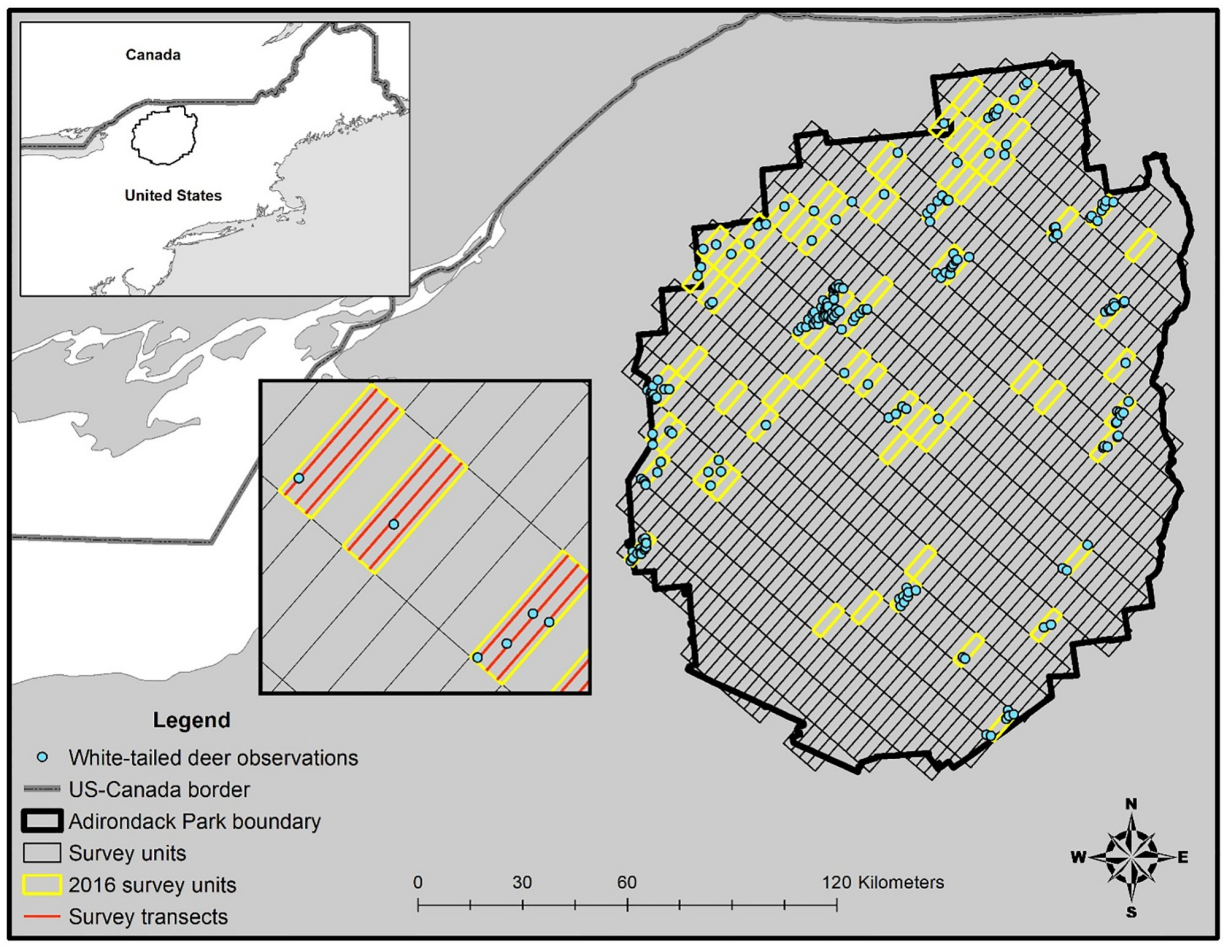
Our study area in the Adirondack Park of northern New York where we conducted aerial surveys of white-tailed deer using distance sampling methods during winter 2016. The rectangular grids represent the 30-km^2^ (10 km x 3 km) survey units available for distance sampling of white-tailed deer and moose. The grids outlined in yellow represent survey units that were randomly surveyed during 2016 to estimate parkwide abundance of white-tailed deer.

The Adirondack Park was located between the temperate and boreal regions of northern New York and consisted of approximately 24,280 km^2^ of private and public lands characterized by mountainous terrain (Fig 1). Elevation in our study area ranged between 30–1,600 m. Mean monthly temperatures in the Park varied between -12 and 6°C and between 20 and 26°C for winter and summer, respectively. Mean monthly precipitation varied between 8–10 cm and annual snowfall between 152 and 356 cm. Predominant vegetation communities in the Park consisted of northern hardwood (~60% of total forest cover) and conifer forest, followed by boreal upland forests and northern swamp. Predominant canopy cover for northern hardwood stands consisted of sugar maple (*Acer saccharum*), red maple (*Acer rubrum*), yellow birch (*Betula alleghaniensis*), white birch (*Betula paperyfera*), and American beech (*Fagus grandifolia*). Boreal stands of red spruce (*Picea rubens*), black spruce (*Picea mariana*), balsam fir (*Abies balsamea*), and eastern hemlock (*Tsuga canadensis*) dominated poorly drained lowland areas, riparian bottomlands, and subalpine forests at elevations above 760 m. More detail of the study area can be found in Kramer et al. [24].

In the Park, publicly owned lands (~48% of the Park) consist of mostly second-growth forest with remnants of original growth because the New York State constitution has protected those areas as “forever wild forest lands” since 1892 [24]. Privately owned lands comprised the remaining areas of the park and were a patchwork of partially cut, uneven-aged forests resulting from timber management [24]. Although timber harvest played a large role in maintaining early seral stages across the Park, the restrictive use of public lands and long rotation age of northern hardwood forests has ensured the dominance of older and thick canopy cover [24–26].

### Survey design

During 2015–2019, monitoring of the low-density moose population in the Park was undertaken via aerial distance sampling [21]. Given the interest in how white-tailed deer may negatively influence moose abundance through competition and disease, deer were also recorded in a winter aerial survey to gain a contemporary, non-harvest-based assessment of their distribution and abundance and compare spatial patterns in moose-deer co-occurrence across the Park.

We partitioned the Park into 10-km × 3-km grid of survey units (N = 784) and surveyed 80 units during winter 2016 (28 Dec 2015–15 Jan 2016; Fig 1). Survey units were oriented at 41.6° to follow the prevailing terrain gradient. Within each survey unit, we surveyed 3, 10-km transects spaced 1 km apart. We sampled survey units once and began surveys at either the northeast or southwest endpoints of a transect in the selected unit, at an altitude of 60 m, and proceeded at a speed of 50–60 kph. Surveys were conducted from either a Bell 430 (Bell Helicopter, Fort Worth, TX, USA) or Robinson 44 (Robinson Helicopter, Torrance, CA, USA) helicopter with a survey crew that included a pilot, a navigator, and 2–3 additional observers with rear-seated observers responsible for recording data. The front passenger (navigator) was equipped with a Windows 8.1 tablet with external antenna (Microsoft Corporation, Redmond, Washington, USA) that was pre-loaded with transect endpoints and displayed and recorded the entire flight path using Quantum GIS [27]. The navigator was also equipped with a handheld GPS unit to save waypoints as a backup. Flights followed all Federal Aviation Administration regulations and were conducted under safe flying conditions (e.g., wind speed <16–24 km/h) after sunrise with consistent snow on the ground. Surveys were conducted for 3–6 hours of flight time/day during the morning through early afternoon.

The survey crew collected distance data on white-tailed deer by directing the pilot off the transect to the location of first detection for each deer group encountered. After a GPS location was acquired directly overhead of the initial sighting point for each white-tailed deer group, the pilot returned to the transect and continued the survey. With the detection locations recorded, we quantified perpendicular distances later using ArcGIS 10.8 [28]. When approached by helicopters, white-tailed deer often flushed and ran short distances, but others also remained bedded or standing. However, the timing and distancing between transect lines ensured fleeing white-tailed deer were unlikely to have been detected more than once in survey units. Although some white-tailed deer likely responded to approaching helicopters by fleeing to cover and escaping detection, CDS allows 60–80% individuals to be missed during surveys and still obtained robust (i.e., unbiased) estimates of the true population size [29].

For each detection, we recorded white-tailed deer group size, time of day, percent cloud cover, and canopy cover type. Canopy cover was classified into 3 types: open (areas with open canopy vegetation or those where canopy was removed due timber harvest), mixed (coniferous and deciduous canopy or conifer only), or hardwood (deciduous canopy). Our survey was approved by the State University of New York College of Environmental Science and Forestry Institutional Animal Care and Use Committee (Protocol Number 140901).

### Statistical analysis

In stage 1 of our analysis, we evaluated the influence of cover type (e.g., open, mixed, hardwood), observer fatigue (e.g., number of preceding survey hours that day), white-tailed deer group size, air temperature (°C measured by embedded sensor on helicopter), and percent cloud cover (measured at start of transect) on deer detection probability using multiple covariate distance sampling (MCDS) using the *Distance* package [30] in R [31] with either a hazard rate or half-normal key function [32]. We ranked models using Akaike’s information criterion (AIC) and goodness-of-fit tests to guide model selection. Competing models were denoted as those within 2 ΔAICs of the top model [33]. After selecting the top ranked MCDS model, we predicted white-tailed deer abundance in each surveyed unit using the Horvitz-Thompson-like estimator [34–36].

In stage 2 of our analysis, we conducted a DSM analysis using the package *dsm* in R [22] in which the spatial variation in distance-corrected abundances of white-tailed deer were modeled using GAMs. We fit a GAM with a Tweedie distribution over the more widely used negative binomial distribution because it allows for more flexible modeling of zeros and extreme values [37]. Coefficient of variation for the abundance estimates of the DSM analyses were obtained through the variance propagation method [38]. When fitting the DSM, this procedure uses an additional random effect term that characterizes uncertainty estimated in the detection function and propagates it through to estimates for the spatial model [22, 23, 39].

For our DSM, we divided each 10-km survey transect (3 transects per 30-km^2^ survey unit) into four 2.5-km segments to measure heterogeneity in land cover types within survey units. We then intersected each survey segment with the GPS locations for each white-tailed deer group detected. Segment-level spatial covariates included a bivariate smooth of geographic coordinates (Universal Transverse Mercator [UTM]) to account for spatially autocorrelated whited-tailed deer detections and land cover types known to influence deer abundance such as distance from agriculture cover (m) [40–43], distance from developed cover (m) [42–45], distance from forest cover (m) [42, 46, 47], distance from shrub cover (m) [43, 48, 49], distance from timber cuts (m) [48, 50], distance from wetland cover (m) [43, 51, 52], distance from water (m) [51, 53], elevation (m) [12, 54, 55], and days with snow cover [56, 57]. Land cover such as agriculture, developed, forest, shrub, timber cuts, wetland, and water were identified using an updated land cover map of the Park for 2016 that combined Landsat 8 satellite imagery (30 m resolution) acquired from the USGS Global Visualization Viewer for imagery, Normalized Difference Vegetation Index, National Land Cover Dataset, the Adirondack Park Agency’s wetlands data, and timber treatment data provided by regional timber companies [24]. We acquired elevation data from a 30-m raster digital elevation model (DEM) [58]. We acquired the number of days with snow cover for each 250-m survey segment from the National Operational Hydrologic Remote Sensing Center’s (NOHSRC) Snow Data Assimilation System Data Products [59]. We considered anthropogenic land cover as landscape features created by humans (e.g., timber cuts, developed cover, and agriculture).

We used principal components analysis (PCA) to compress our highly dimensional spatial dataset into the dominant, underlying gradients of variation (principal components [PCs]). We used the latent root criterion (PCs with eigenvalues ≥1) as a stopping rule to determine the number of significant PCs to retain and interpret [60]. We then based our interpretation of each PC on those variables with loadings ≥0.40 or ≤-0.40 and placed most emphasis on those with loadings ≥0.60 or ≤-0.60 [60]. We used the variables with the strongest loadings to interpret the ecological meaning of each PC. The PCs were then used as indicators of land cover complexes and variables of landcover gradients existing in our study areas, in which white-tailed deer abundance either increased or decreased with the value of each of the latent environmental variables.

For our GAM models, we used restricted maximum likelihood (REML) to optimize parameter estimates along with a thin-plate spline shrinkage approach to modify the smoothing penalty for model selection purposes [61]. In addition to environmental predictors, we included a UTM bivariate term to provide a spatial estimator, which acted as a proxy for unmeasured variables that influence abundance not accounted for by the other predictors. We explored all possible subsets of the 4 predictor variables including the null model as candidate models and evaluated model sets using Akaike’s information criterion (AIC) to guide model selection. In addition to AIC, we also compared estimates among models by the ability to include informative covariates and produce reliable population estimates (i.e., coefficient of variation [CV]). We considered the model with the lowest AIC as the best approximating model. However, when model sets had ≥2 models that were within 2 ΔAIC of the top model, we selected the most parsimonious model.

To map predicted white-tailed deer density, we created a grid of 1-km^2^ cells across the Park (*n* = 23,998 cells), calculated UTM coordinates at cell midpoints, and extracted spatial covariates for each cell. As noted by Miller et al. [22], prediction grid cells that are smaller than the resolution of the spatially referenced data have no effect on abundance or density estimates. Applying the selected model to that grid enabled a spatially explicit estimate of deer abundance, density (deer/km^2^), and coefficient of variation [22]. Total white-tailed deer abundance for the Park was then obtained by summing predicted deer abundance across all relevant cells. To correlate winter densities of white-tailed deer and moose in the Park, we used a Spearman rank-order correlation to compare our deer density surface map to a moose density surface map previously derived from the same aerial surveys [21].

## Results

During 10 survey flights, we recorded 200 observations of white-tailed deer groups after surveying 2,400 km of transects in the Park, comprising a total of 645 individuals (0.083 detections/km). Deer group size ranged 1–30 animals and averaged 3.21 (SD = 2.89; *n* = 200 groups). The maximum distance to which a deer group was observed was 414 m, but observations were truncated to 350 m when fitting the detection function (yielding *n* = 193 groups). We detected no difference among our top 5 ranked detection models and therefore selected the most parsimonious one which included a hazard rate function with canopy cover as the only covariate indicating that white-tailed deer detection probability was greatest under hardwood and open cover compared to those under conifer cover (Tables 1 and 2). On average, survey teams observed ~58% of the deer estimated to occur along survey transects ($\hat{p}=0.58\pm 0.03$ SE; Table 1).

**Table 1:** Summary of the top 6 candidate detection function models with hazard rate key functions for white-tailed deer in the Adirondack Park, 2016. Indicated for each model are the model covariates, number of estimated parameters (*K*), Cramer von Miser goodness of fit *P*-value, average probability of detection ($\hat{p}$) with standard error (SE), and ΔAIC comparing models.

Model	*K*	C-vM *P*-value	$\hat{p}$	SE	ΔAIC
Canopy cover + cloud cover	4	0.703	0.66	0.030	0.00
Canopy cover	3	0.612	0.66	0.030	0.77
Canopy cover + temperature + cloud cover	5	0.630	0.67	0.029	1.17
Canopy cover + group size + cloud cover	5	0.711	0.66	0.030	1.79
Canopy cover + fatigue + cloud cover	5	0.721	0.66	0.030	1.92
Canopy cover + group size	4	0.629	0.66	0.030	2.04

**Table 2:** Parameter estimates for the most parsimonious detection model for surveying white-tailed deer via helicopter during 2016 in Adirondack Park, New York. Cover was a categorical variable and, when significant in a model, hardwood cover served as the reference category. Shown are β coefficients and standard error (SE).

Model	Key function	Parameter	β	SE
Canopy cover	Hazard rate	Intercept	-1.680	0.094
		Mixed	0.054	0.112
		Open	0.321	0.112

The first 3 principal components (PC1, PC2, and PC3) explained 31.6%, 23.2%, and 11.9% of the cumulative variation in our land cover predictors, respectively and were the only PC scores with eigenvalues ≥1 (Table 3). Since the first 3 axes explained 66.8% of the total variance, we deemed the 3-dimensional solution adequate. PC consisted of positive loadings for elevation, distance from agriculture cover, distance from developed cover, distance from timber cuts, and number of days with snow cover—these variables contributed 97.4% of PC1’s quality of representation (Table 3). Therefore, we interpreted decreasing PC1 scores as land cover that provided thermal cover (areas with lower elevation and fewer days with snow cover) and food resources (decreased distances from agriculture cover, developed cover, and timber cuts). PC2 consisted of large positive loadings for distances from forest cover, wetland cover, and shrub cover—contributing 91.0% of PC2’s quality of representation (Table 3). PC3 consisted of a single large positive loading for distance from water and contributed 84.4% of PC3’s quality of representation (Table 3). Collectively, the PC scores indicate that once PC1 accounted for anthropogenic land cover, elevation, and number of days with snow cover, PC2 and PC3 accounted for preserved state land and bodies of water, respectively.

**Table 3:** Factor loadings and percent contribution for each of the top 3 principal components (PC) for land cover measured across the Adirondack Park, New York.

Land cover	Principal component 1 (Thermal cover and forage)		Principal component 2 (Preserved state land)		Principal component 3 (Distance to bodies of water)	
	Loading	% Contribution	Loading	% Contribution	Loading	% Contribution
Agriculture^1^	**0.67**	**15.43**	0.00	0.00	-0.22	4.48
Developed^1^	**0.76**	**20.05**	0.30	4.35	-0.16	2.22
Forest^1^	-0.29	2.87	**0.85**	**34.87**	-0.14	1.75
Shrub^1^	-0.06	0.13	**0.67**	**21.79**	0.19	3.40
Timber^1^	**0.69**	**16.63**	0.38	6.94	-0.05	0.20
Wetland^1^	-0.12	0.47	**0.80**	**31.43**	-0.12	4.20
Water^1^	0.15	0.82	-0.06	0.16	**0.94**	**82.38**
Elevation	**0.83**	**23.66**	-0.09	0.40	0.19	3.34
Snow^2^	**0.76**	**19.96**	-0.04	0.06	-0.19	0.03
Eigenvalue	2.88		2.06		1.08	
% of total variance	31.96		22.88		11.99	

^1^Distance-based variables

^2^Number of days with snow cover

Also included are eigenvalue, percent of total variance explained, and descriptions of principal components. Interpretation of PCs are parenthesized. Variables with significant loadings shown in bold.

**Fig 2:**
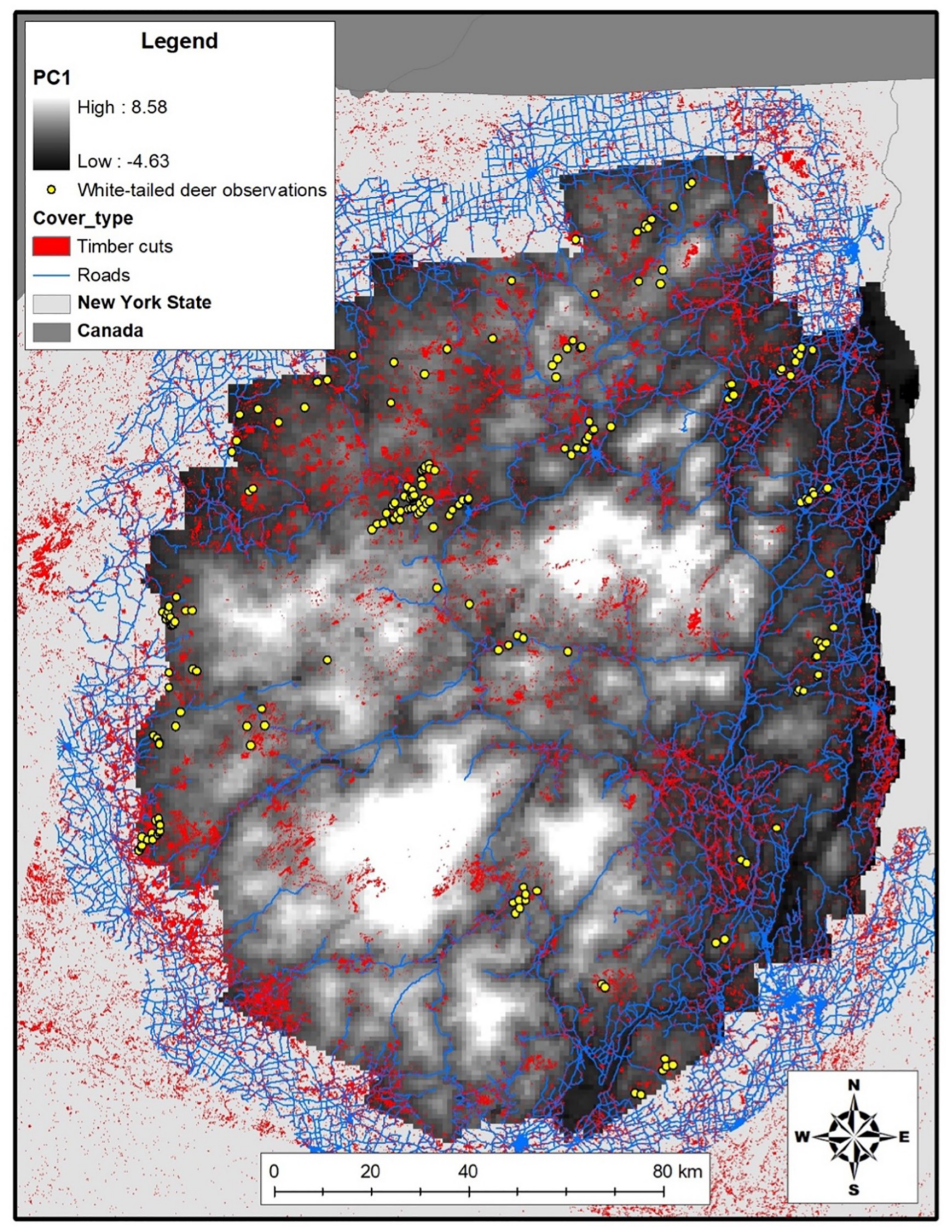
Observations of white-tailed deer made during aerial surveys (yellow circles), roads (blue lines), and timber cuts (red polygons) overlaid on a surface map of PC1 representing thermal cover and foraging areas for deer across the Adirondack Park, New York, USA, during winter 2015–2016. Low PC1 values represent areas with low elevation, decreasing days with snow cover, and increasing proximity to timber cuts, developed cover, and agriculture.

**Fig 3:**
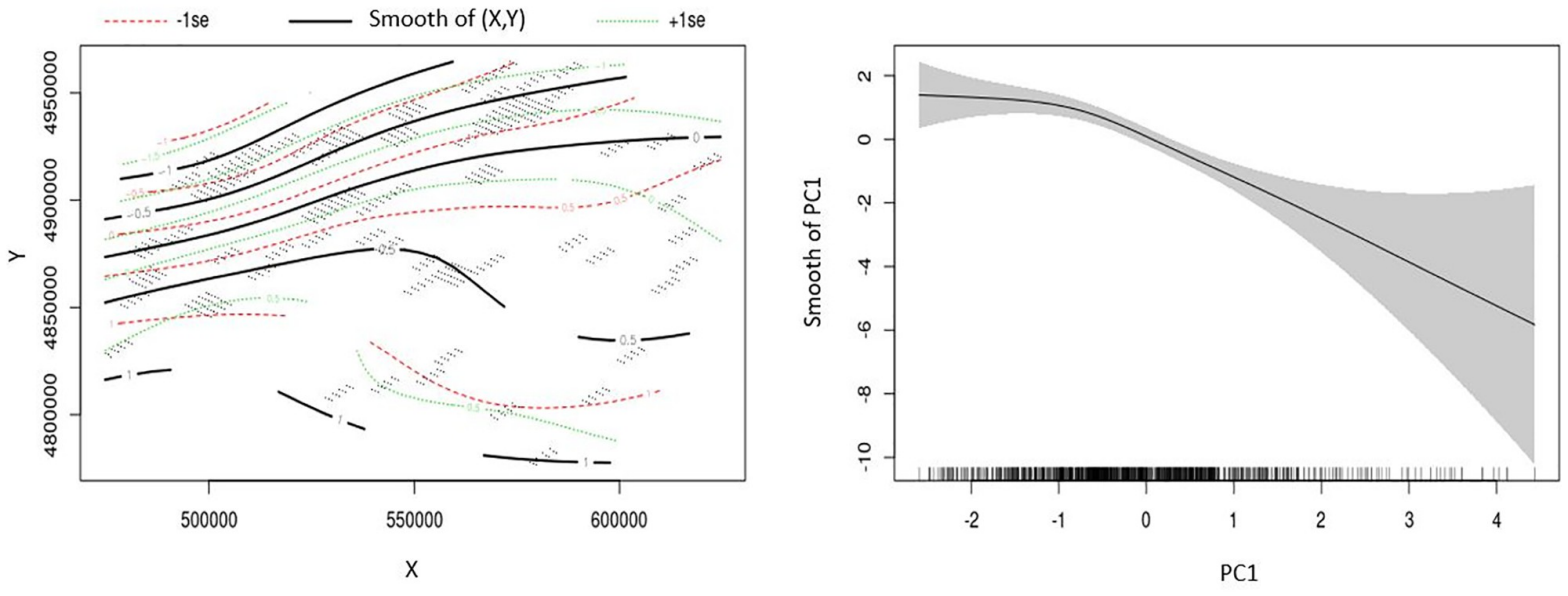
Partial effects of statistically significant predictors on winter abundance of white-tailed deer in the Adirondack Park, New York during 2016 according to the best fitting generalized additive model (GAM). A) Two‐dimensional plot of the bivariate geographic coordinates. The solid black lines represent the smooth functions. The locations of each 2.5-km^2^ survey segment are plotted as small dots. The dotted red and green lines represent −1 standard error and +1 standard error, respectively. The number on the lines indicate whether geographic coordinates had a positive effect (e.g., +1), a negative effect (e.g., −1), or were neutral (0) on deer abundance. B) Smooth function for the mean effect of the first principal component (PC1) (thermal cover and anthropogenic cover). The shaded area represents the 95% confidence intervals for the mean effect. The rug ticks at the bottom of the plot indicate the coverage of the range of values of each variable in the units surveyed. The number in parentheses on the y-axis indicate the effective degrees of freedom (a measure of flexibility) of each term.

**Fig 4:**
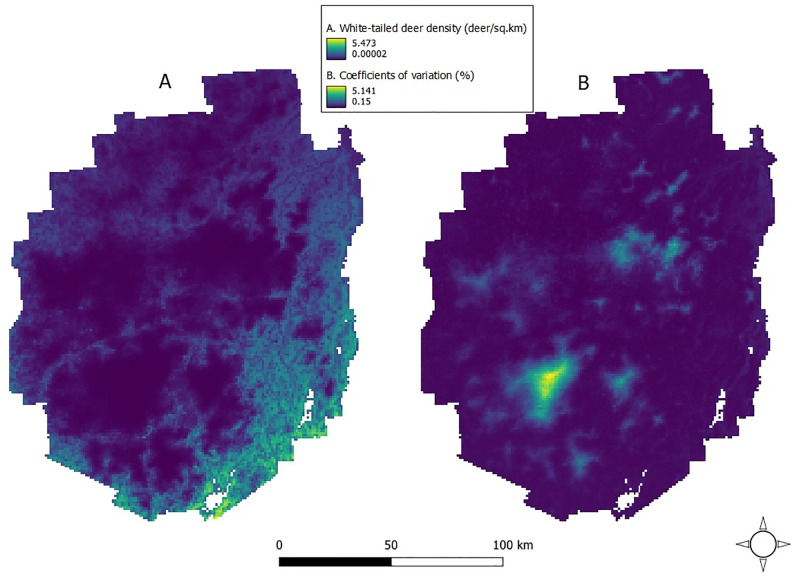
A) Predicted white-tailed deer density (individuals/km^2^) for 1 km^2^ grids across the Adirondack Park, New York, USA, from a density surface model using distance sampling data via aerial surveys conducted during winter 2016. B) Map of the coefficients of variation for the selected model. High uncertainty occurs in areas where there was low sampling effort (Fig 1).

**Fig 5:**
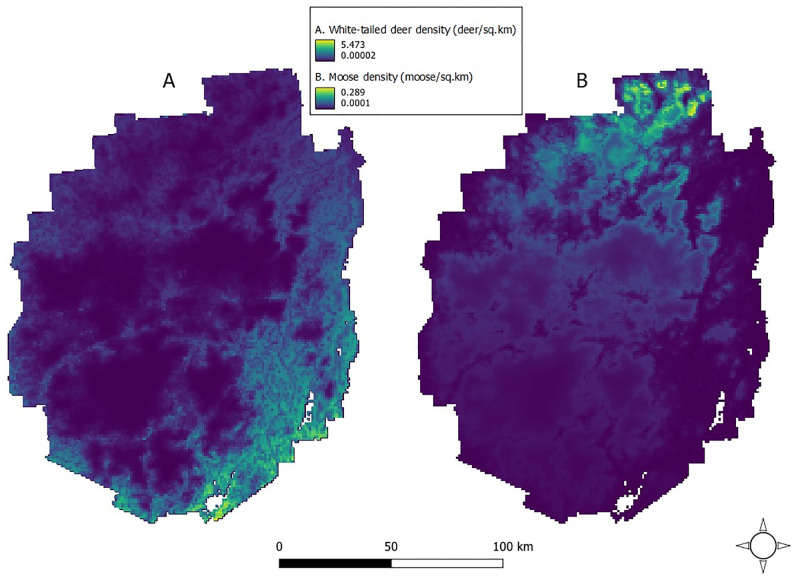
A) Predicted white-tailed deer density (individuals/km^2^) for 1 km^2^ grids across the Adirondack Park, New York, USA, from a density surface model using distance sampling data via aerial surveys conducted during winter 2016. B) Predicted moose density (individuals/km^2^) for 1-km^2^ grids across the Adirondack Park, New York, USA, from a density surface model using distance sampling data via aerial surveys, 2016–2019 [21].

According to AIC, our best GAM model indicated that geographic coordinates and PC1 to be informative for predicting local white-tailed deer density (Table 3, Fig 2). White-tailed deer abundance was predicted to decrease in areas with increasing elevation and number of days with snow cover as well as areas with increasing distance from timber cuts, developed cover, and agriculture (PC1; Tables 4 and 5, Fig 3). Overall white-tailed deer density for the Park was estimated from the GAM to be 0.70 deer/km^2^ (95% CI 0.50–0.97). Our DSM approach revealed local densities ranging 0.00–5.47 deer/km^2^ with specific regional concentrations (Fig 4). We estimated 16,352 deer (95% CI 11,762–22,734) across the Park and achieved a 16.8% CV, which fell below the desired 20% threshold (see Buckland et al. 2008 [29]). Spearman’s ρ = -0.61 was estimated between the relative densities of our white-tailed deer and those reported previously for the Park’s moose [21] (Fig 5).

**Table 4:** Top GAM models and the null model for white-tailed deer abundances in the Adirondack Park, New York, USA. For each area and period, we provide the estimated deer abundance (N), 95% confidence interval (CI), coefficient of variation (CV), and ΔAIC.

Model	Estimated deer abundance ($\hat{N}$)	95% CI for $\hat{N}$	CV	ΔAIC
Geographic coordinates + PC1^1^	16,352	11,762–22,734	0.169	0.00
Geographic coordinates + PC1 + PC3^2^	16,801	12,121–23,287	0.168	3.48
Geographic coordinates + PC1 + PC2^3^ + PC3	16,802	12,121–22,290	0.168	3.48
PC1 + PC3	12,105	9,170–15,979	0.142	19.43
PC1	11,905	9,082–15,603	0.139	22.28
Geographic coordinates	15,771	11,723–21,209	0.152	47.82
Null	11,455	9,100–14,419	0.112	72.73

^1^Thermal cover and forage

^2^Distance from large bodies of water

^3^Preserved state land

**Table 5:** Summary of the generalized additive model describing factors influencing white-tailed deer abundance in Adirondack Park, New York, USA, 2016. We present the estimated degrees of freedom (edf), reference degrees of freedom (Ref.df), *F* statistic, and *P*-value for each term.

	edf	Ref.df	*F*	*P*-value
Geographic coordinates	4.985	9	3.422	<0.001
PC1^1^	2.483	9	4.971	<0.001

^1^Thermal cover and forage

## Discussion

Our DSM approach allowed us to assess the spatial variation in the abundance of white-tailed deer across the Park during winter using environmental predictors to detect significant changes in local abundances. Unlike the CDS methods, DSM use observations to model the effect of predictor variables on abundance. Therefore, these methods can improve management strategies for white-tailed deer, identifying primary land cover (e.g., timber cuts and roads) and environmental conditions (e.g., snow conditions) influencing white-tailed deer abundance. Our abundance estimate and density surface map provide valuable insights into several relevant areas for the management of white-tailed deer in the Park.

Annual hunter harvests have been the primary source of information regarding the white-tailed deer population in the Park. For example, prior to our winter survey, the 2015 white-tailed deer hunting season (27 Sep–13 Dec 2015) resulted in 8,566 deer harvested (0.35 deer/km^2^) across the 7 WMUs (5C, 5F, 5G, 5H, 5J, 6F, 6J) that made up the study area [62]. Given that our selected model estimated an overall abundance of 16,352 white-tailed deer (0.70 deer/km^2^) across the Park immediately following the 2015 hunting season, a simple intuitive interpretation of both datasets suggests that approximately 24,918 white-tailed deer (1.03 deer/km^2^) may have occupied the Park prior to the 2015 deer hunting season by which up to 34.4% of the population was harvested by hunters. Furthermore, the greatest density of white-tailed deer occurred in the eastern and southern regions of the Park (Fig 4) which coincided with the wildlife management units (WMUs) where deer harvest was greatest in the Park.

Much of the predictive power from our selected model was derived from the bivariate spatial predictor (geographic coordinates) and PC1 (elevation, days with snow cover, and distances from timber cuts, developed cover, and agriculture). Spatial variation in white-tailed deer was most strongly correlated with geographic coordinates and, given the large influence that geographic coordinates had in our model, the bivariate spatial predictor likely accounted for important unmodelled environmental and social characteristics not captured by PC1. For example, geographic coordinates may have captured the latitudinal influence that the Park’s transition between northern-temperature and southern boreal biomes had on white-tailed deer abundance and distribution. Additionally, white-tailed deer have historically occupied the peripheral areas of the Park and geographic coordinates may have represented proximity of deer to source populations adjacent to the Park. Furthermore, geographic coordinates accounted for the spatial autocorrelated distribution of white-tailed deer that could be attributed to yarding behavior and other social characteristics of deer important for reducing winter mortality.

Regardless, our objective for using a DSM was to identify and describe where white-tailed deer densities were greatest in the Park. The correlation between white-tailed deer abundance and PC1 indicated that abundance increased in areas characterized by fewer days with snow cover, lower elevation, and proximity to timber cuts, developed cover, and agriculture. This was characteristic of areas along the Park’s southern and eastern borders where we observed the greatest white-tailed deer densities. Conversely, white-tailed deer densities were lowest in the central and northern areas of the Park that experienced increasing elevation and days with snow cover and were farther from timber cuts, developed cover, and agriculture. The remaining white-tailed deer in the Park occurred at moderate densities in narrow, linear distributions that highlighted potential movement corridors for deer in the region, as these potential movement corridors experienced less snow cover and were closer to roads and timber cuts than the surrounding areas with lower deer density.

Our findings support Hurst and Porter’s [12] conclusion that snow cover influenced white-tailed deer movements during winter, roads and waterways facilitated their movements among winter yards, and forest regeneration associated with timber cuts provided them forage. Reduced snow cover and increased growing seasons caused by warming winters will likely increase white-tailed deer abundance and distribution in the Park, as deep snows increased winter mortality for deer and contained them to specific winter yards in peripheral areas of the Park [12]. As climate becomes more favorable, white-tailed deer will experience improved winter survival rates, body conditions, and dispersal capabilities that will increase their abundance and allow them to spread into other areas of the Park during milder winters.

This should be expected as climate change and anthropogenic land use are known to facilitate the range expansion of white-tailed deer [3, 4, 63]. Dawe and Boutin [4] reported that climate, as an index of winter severity, was the most important factor determining white-tailed deer distribution at northern latitudes whereas anthropogenic land use was determined to be a secondary factor. Our findings align with these previous studies, as white-tailed deer density in the Park was positively correlated with low elevation, fewer days with snow cover, and greater anthropogenic land cover. White-tailed deer use some land cover, such as roads and riparian areas, as movement and dispersal corridors [44, 45, 64, 65] while exploiting timber cuts, albeit a land cover type that is less stable spatially in terms of winter forage availability than are roads and riparian areas [10, 66, 67]. Despite timber cuts accounting for only ~9% of the Park’s area, white-tailed deer aggregated in and around timber cuts suggesting that local distribution of deer during winter were influenced by food resources provided by regenerative growth. Consequently, improved food resources allow white-tailed deer to enter harsher periods of the winter with better body conditions, improves their winter survival, and permits females to accumulate fat during winter to meet the energetic demands of lactation and reproduction during spring [67–69].

Although over-browsing by white-tailed deer was documented to reduce forest regeneration, sustainability, and health in the region [20, 70–72] and is a serious concern for wildlife managers, parasite-mediated competition between deer and moose is the primary concern in the Park [21]. For example, white-tailed deer density in areas of the Park’s southeastern region approach or exceed the >4 deer/km^2^ where meningeal worm is likely to pose as threat to moose [73, 74] and in the northern region moose density approaches the lowest densities (range = 0.29–3.1 moose/km^2^) reported for populations that experienced outbreaks in winter tick (*Dermacentor albipictus*) [75–77]. Currently, white-tailed deer and moose exhibit some spatial segregation in the Park. According to our predictions and distribution maps, white-tailed deer density was ≤2 individuals/km^2^ for 91.2% of the Park with some of the lowest deer densities (0–0.25 deer/km^2^) occurring in the northern region where moose densities were reported to be the highest [21] (Fig 5). Furthermore, we found a moderate negative correlation (ρ = -0.61) between white-tailed deer density and moose density throughout the Park. However, climate-favored expansion of white-tailed deer into regions of the Park that were historically moose-only may reduce reproduction and survival of moose via parasite transmission.

White-tailed deer abundance was positively correlated with timber cuts, as was moose abundance [21], but deer abundance responded positively to areas near roads and that experienced fewer days with snow cover. This suggests that snow conditions may serve as an important mechanism promoting co-occurrence in the region by facilitating differential accessibility to patchy resources by white-tailed deer and moose resulting in spatial segregation of both species at local scales. However, changes to white-tailed deer distributions in the Park caused by milder winters may allow greater densities of deer to persist throughout the region, leading to detrimental effects on moose through food competition and disease spread. In particular, anthropogenic cover such as residential areas may inflate deer densities encountering each other and these could allow spread of parasites or prions (e.g., chronic wasting disease [CWD]) [65, 78, 79]. Road networks across the Park would then serve as disease transmission corridors because deer routinely use roads as movement corridors into areas occupied by moose [12, 44, 45]. Shared habitat and food resources could spread parasites and CWD to moose. Therefore, correlating land cover with the distribution and abundance of white-tailed deer and moose will shed light on which factors facilitate contact, competition, and co-occurrence between deer and moose at different scales and will require a more sophisticated suite of predictor variables than those used in this study.

## Conclusions

Since 1954, white-tailed deer populations in the Park have been monitored through harvest records at the spatial scale of WMUs [80]. However, harvest records are insufficient for quantifying relationships between white-tailed deer abundance and land cover characteristics that are essential for predicting future changes in the distribution of deer as the climate becomes more favorable to them. Aerial surveys combined with CDS and DSM resolves this problem by allowing wildlife managers to readily track change in the distribution and abundance of deer over time and across spatial scales independent of WMUs. Therefore, DSMs can play an essential role in developing future monitoring and management strategies with contemporary, non-harvest-based assessment of white-tailed deer distribution and abundance. Indeed, distribution and density maps resulting from DSMs enables easily interpretable results and will ease the access to scientific information essential for management plans. This will allow wildlife managers to devise strategies for mitigating key threats to moose in the region, such as epizootics [81–83]. Furthermore, when disentangling factors responsible for the observed distributions of white-tailed deer, future surveys and analyses should consider density of potential competitors (e.g., moose), disturbance and mortality factors, prevalence of pathogens, effects of climate change (e.g., winter severity), and summer forage availability.

## Supporting information

S1 Data (CSV)

S2 Data (CSV)

S3 Data (CSV)

S4 Data (CSV)
